# Applied Circular Dichroism: A Facile Spectroscopic Tool for Configurational Assignment and Determination of Enantiopurity

**DOI:** 10.1155/2015/865605

**Published:** 2015-01-29

**Authors:** Macduff O. Okuom, Raychelle Burks, Crysta Naylor, Andrea E. Holmes

**Affiliations:** Department of Chemistry, Doane College, 1014 Boswell Avenue, Crete, NE 68333, USA

## Abstract

In order to determine if electronic circular dichroism (ECD) is a good tool for the qualitative evaluation of absolute configuration and enantiopurity in the absence of chiral high performance liquid chromatography (HPLC), ECD studies were performed on several prescriptions and over-the-counter drugs. Cotton effects (CE) were observed for both S and R isomers between 200 and 300 nm. For the drugs examined in this study, the S isomers showed a negative CE, while the R isomers displayed a positive CE. The ECD spectra of both enantiomers were nearly mirror images, with the amplitude proportional to the enantiopurity. Plotting the differential extinction coefficient (Δ*ε*) versus enantiopurity at the wavelength of maximum amplitude yielded linear standard curves with coefficients of determination (*R* ^2^) greater than 97% for both isomers in all cases. As expected, Equate, Advil, and Motrin, each containing a racemic mixture of ibuprofen, yielded no chiroptical signal. ECD spectra of Suphedrine and Sudafed revealed that each of them is rich in 1S,2S-pseudoephedrine, while the analysis of Equate vapor inhaler is rich in R-methamphetamine.

## 1. Introduction

Regulations of the United States Food and Drug Administration (FDA) [1] require that the properties of stereoisomers in modern drugs be known [2, 3]. Although some pharmaceuticals contain racemic mixtures, such as Advil that contains both enantiomers of ibuprofen, the emphasis on enantiopure compounds being more effective than their racemic counterparts has dominated the field of chiral separation methods [3, 4]. In the pharmaceutical industry, determination of enantiopurity is mainly done using high performance liquid chromatography (HPLC) coupled with chiral columns [5, 6]. In addition to the determination of enantiopurity, it is important to ascertain stereochemical assignments and assess the interconversion of stereoisomers [6, 7]. The isolation of enantiomers and the assessment of optical purity still remain a challenge [6, 8, 9]. Commonly used methods for the determination of absolute configuration include electronic chiroptical spectroscopic techniques such as optical rotatory dispersion [10, 11], polarimetry [12], circularly polarized luminescence [10], fluorescence-detected circular dichroism [13], and vibrational circular dichroism (VCD) [14]. Other nonchiroptical methods include nuclear magnetic resonance (NMR), mass spectrometry [15–18], X-ray crystallography [19], and chiral HPLC [20, 21].

The main purpose of this work is to show that ECD can be used as a tool to determine enantiopurity of pharmaceuticals [22] when more sophisticated methods are not readily available, such as the ones discussed in the literature [23–25]. One advantage of ECD is that it can rapidly discriminate enantiomers, as well as diastereomers, and has the ability to determine the composition of racemic mixtures [26]. ECD requires millimolar or lesser concentrations, making it well-suited for use when the quantity of the sample is limited. We explored polarimetry, but this method is well known for high analyte concentrations and large sample volume [27, 28].

**Figure 1:**
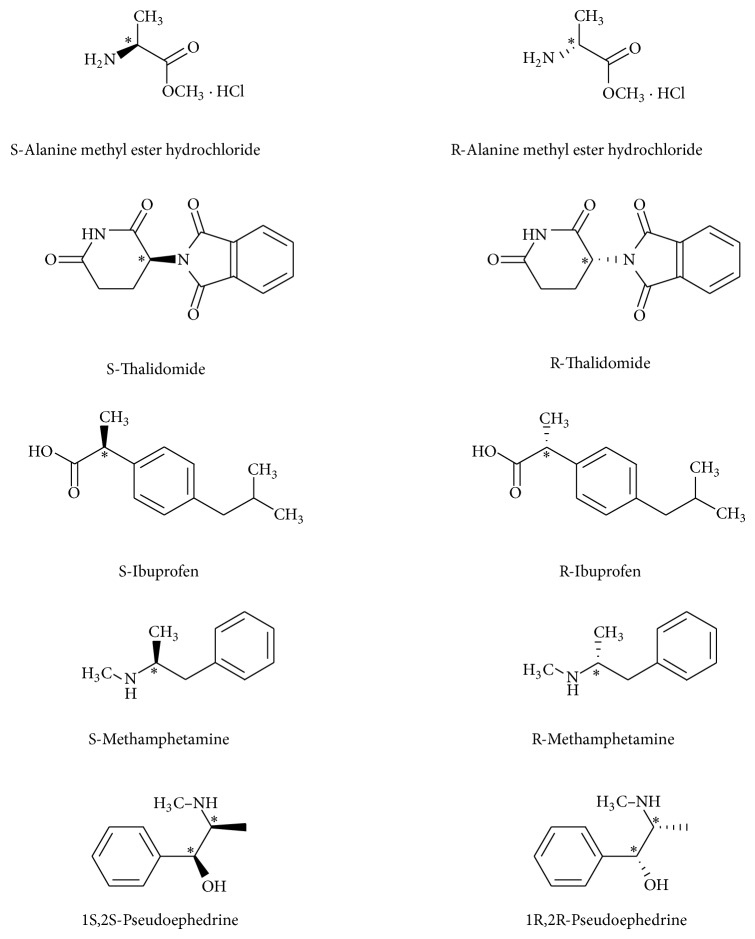
Enantiomers of alanine methyl ester hydrochloride, thalidomide, ibuprofen, methamphetamine, and pseudoephedrine. The chiral center for each enantiomer has been labeled by an asterisk ( ^*^).

We explored the possibility of using ECD as a simple tool for semiquantitative determination of enantiopurity of alanine, thalidomide, ibuprofen, methamphetamine, and pseudoephedrine (Figure 1). Alanine was selected because both enantiomers are readily available and inexpensive. Thalidomide was used because of its pharmaceutical relevance [29, 30], while ibuprofen was used because it is commercially prepared as a racemic mixture in products such as Motrin and Advil. Sudafed was used because it is commonly used as an over-the-counter (OTC) nasal decongestant and contains two chiral centers, (1S,2S)-pseudoephedrine [31]. S-Methamphetamine was used for this study because it is classified in the US as a Schedule II drug. R-Methamphetamine, in contrast, is not classified as a controlled substance in the US and has the ability to constrict blood vessels. It is an ingredient in selecting OTC nasal decongestants such as Vicks vapor inhaler [32].

## 2. Materials and Methods

The following items were purchased from Sigma-Aldrich, (Milwaukee, Wisconsin): L-alanine methyl ester hydrochloride (L-alanine), D-alanine methyl ester hydrochloride (D-alanine), L-thalidomide, D-thalidomide, S-ibuprofen, deoxyephedrine (R-methamphetamine), and methamphetamine hydrochloride (S-methamphetamine). R-ibuprofen was obtained from Santa Cruz Biotechnology (Santa Cruz, California). 1S,2S- and 1R,2R-pseudoephedrine were obtained from Fisher Scientific (Pittsburgh, Pennsylvania).

Stock solutions of R- and S-alanine, R- and S-ibuprofen, and R- and S-methamphetamine (3.0 mM in methanol) were used to prepare 10-fold dilutions of analytes. Stock solutions of R- and S-thalidomide and 1R,2R-pseudoephedrine and 1S,2S-pseudoephedrine (0.30 mM, methanol) solutions were used to prepare 10-fold dilutions of (0.030 mM) of analytes.

The following were purchased from the local retail store: Equate ibuprofen, Advil, and Motrin (each with 200 mg of ibuprofen); Sudafed and Suphedrine (each with 120 mg of pseudoephedrine); Equate vapor inhaler (with 198 mg of R-methamphetamine).

Using a razor blade, the coating was removed from one pill of each of the OTC ibuprofen and then crushed using a pestle and mortar. The crushed solids were dissolved in methanol (3.0 mM). Insoluble material was isolated and removed by centrifugation. The sponge inside the Equate vapor inhaler tube was removed and soaked in 50 mL of methanol for 1 h to extract the active ingredient, R-methamphetamine (21.0 mM). Sample solutions of 0.3 mM methamphetamine were made from this stock solution in methanol.

A JASCO J-815 CD spectrometer was used for sample analysis, employing a 1 cm quartz cuvette. Instrument conditions were as follows: room temperature setting, bandwidth of 2 nm, response time of 1 second, standard sensitivity, wavelength range of 300–200 nm, data pitch of 1 nm, scanning speed of 50 nm/min, and a four-scan accumulation (averaged at end). The CD spectra of all alanine, ibuprofen, and pseudoephedrine samples were recorded in methanol at molar concentrations of 0.30 mM at various enantiopurities (0–100% at 10% increments). The thalidomide enantiomers were investigated at 0.030 mM concentrations under the same CD conditions. The spectra for the methamphetamine samples were normalized by subtracting a spectrum of methanol under the same conditions. Data analysis and the determination standard error were done using Microsoft Excel.

## 3. Results and Discussion

**Figure 2:**
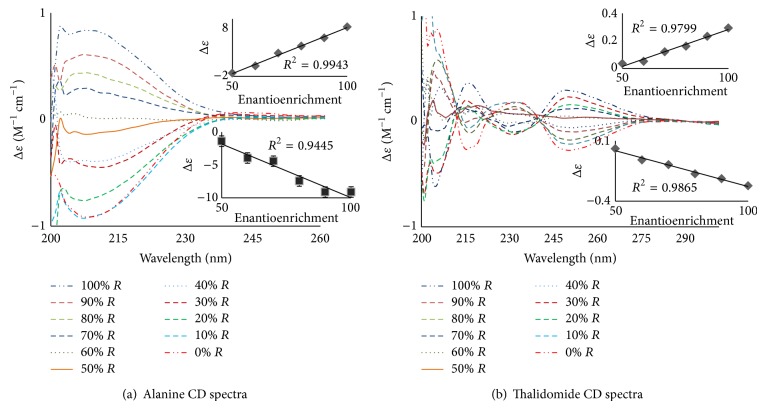
CD standard curves of not-over-the-counter available substances: (a) alanine and (b) thalidomide. The spectra were measured for solutions of increasing concentration of the R-enantiomer from 0 to 100% as shown in the legend for both compounds. Insets are linear regression graphs showing between enantioenrichments versus Δ*ε* at the wavelength of maximum absorption. All analytes were dissolved in methanol at 0.3 mM concentrations. All readings were determined at room temperature.

**Figure 3:**
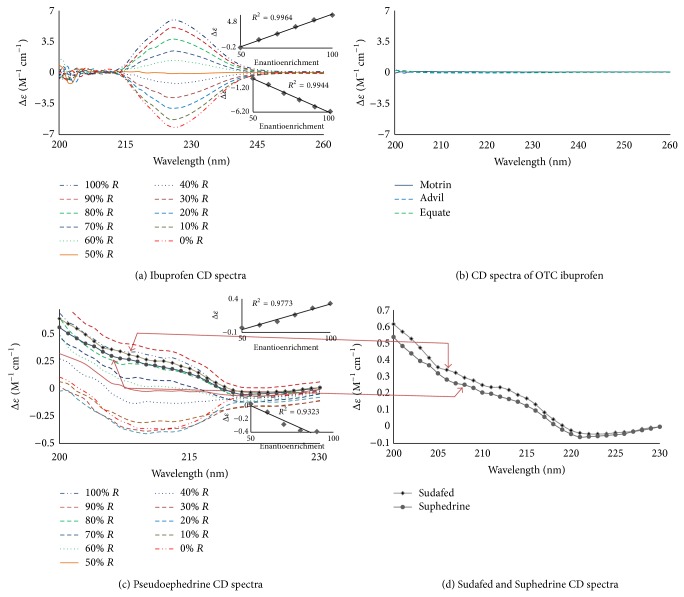
CD spectra of varying enantioenrichment of the R-enantiomer (0–100%) for OTC available substances: (a) ibuprofen, (c) pseudoephedrine, and the OTC drugs of (b) Motrin, Advil and Equate ibuprofen, (d) Sudafed and Suphedrine at 0.3 mM concentration in methanol with a corresponding linear regression between enantioenrichment and Δ*ε* at the wavelength of maximum absorption shown in the inset. All readings were taken at room temperature.

**Figure 4:**
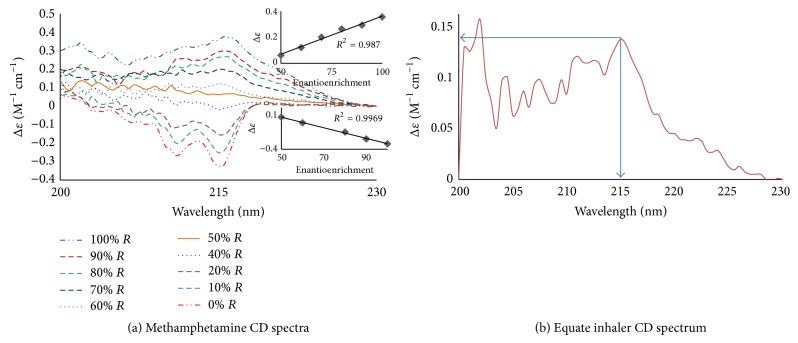
CD spectra for (a) varying enantioenrichment of the R enantiomer (0–100%) of 0.3 mM methamphetamine in methanol. (b) 0.3 mM Equate inhaler in methanol.

Figure 2 shows the ECD spectra for aniline and thalidomide.  Figure 2(a) shows the ECD spectra for alanine. The R enantiomer has a positive CE, while the S enantiomer has a negative CE centered between 200 and 240 nm, with a maximum amplitude of 210 nm. The 100% S-enantiomer shows an amplitude of Δ*ε* = −0.909 M^−1^ cm^−1^, and the 100% R enantiomer shows an amplitude of Δ*ε* = +0.832 M^−1^ cm^−1^. As the enantioenrichment decreases, the amplitude decreases until it is optically inactive (Δ*ε* = 0 M^−1^ cm^−1^ at 0% enantioenrichment). Similar results were observed for thalidomide (Figure 2(b)), ibuprofen, pseudoephedrine, and methamphetamine (Figures 3(a), 3(c), 3(e), and 4(a), resp.). Predominance by the R isomer gives a positive CE, while the S isomer displays a negative CE. The linear relationships between the enantioenrichment and Δ*ε* are shown as insets and demonstrate excellent coefficients of determination (greater than 94%, the standard error in the linear regression lines are shown as error bars).

In order to determine the enantiopurity of the over-the-counter (OTC) drugs, ECD spectra were obtained for 0.30 mM methanolic solutions of Equate ibuprofen, Motrin, Advil, Sudafed, Suphedrin, and Equate vapor inhaler. No chiroptical signals were observed in the CD spectra for each of the OTC ibuprofen products (Figure 3(b)), which was expected as these substances are racemic and thus optically inactive.

Pseudoephedrine that has two chiral centers and the CD spectra were more complicated (Figure 3(c)). However, it was determined that Sudafed and Suphedrine showed an enantioenrichment of the R isomer of approximately 65% (Figure 3(c)), when their curves were compared to the pure pseudoephedrine curves in Figure 3(b).

The Equate vapor inhaler containing R-methamphetamine showed a Δ*ε* of approximately 0.135 M^−1^ cm^−1^ at 215 nm (Figure 4(a)). Extrapolating this value to the standard curve of methamphetamine (Figure 4(b)), it can be estimated that the Equate vapor inhaler is at least 65% enantioenrichment of R-methamphetamine.

The enantioenrichment of 65% of Sudafed, Suphedrine, and Equate vapor inhaler seems to indicate that the OTC drugs are not quite enantiopure. While the apparent loss of enantiopurity for this result remains unclear, the possible presence of impurities leading to a complex mixture may contribute to this finding.

## 4. Conclusion

ECD spectroscopy is an easy and quick semiquantitative method for the determination of absolute configuration and enantiopurity of chiral pharmaceuticals. We demonstrated that the method does not require high sample concentrations, is user friendly, and may be used as an alternative method when chiral HPLC or HPLC coupled with ECD is not available.
